# Identification keys to the *Anopheles* mosquitoes of South America (Diptera: Culicidae). IV. Adult females

**DOI:** 10.1186/s13071-020-04301-0

**Published:** 2020-11-18

**Authors:** Maria Anice Mureb Sallum, Ranulfo González Obando, Nancy Carrejo, Richard C. Wilkerson

**Affiliations:** 1 Departamento de Epidemiologia, Faculdade de Saúde Pública, Universidade de São Paulo, Avenida Doutor Arnaldo 715, São Paulo, São Paulo CEP01246-904 Brazil; 2 Departamento de Biología, Universidad del Valle, A.A 25360 Cali, Colombia; 3 Department of Entomology, Smithsonian Institution, National Museum of Natural History (NMNH), Washington, DC 20560 USA; 4 Walter Reed Biosystematics Unit, Smithsonian Institution Museum Support Center, 4210 Silver Hill Rd., Suitland, MD 20746 USA; 5 Walter Reed Army Institute of Research, 503 Robert Grant Avenue, Silver Spring, MD 20910 USA

**Keywords:** *Anopheles*, Illustrated key, Morphology, Identification, South America

## Abstract

**Background:**

Morphological identification of adult females of described species of the genus *Anopheles* Meigen, 1818 in South America is problematic, but necessary due to their differing roles in the transmission of human malaria. The increase in the number of species complexes uncovered by molecular taxonomy challenges accurate identification using morphology. In addition, the majority of newly discovered species have not been formally described and in some cases the identities of the nominotypical species of species complexes have not been resolved. Here, we provide an up-to-date key to identify Neotropical *Anopheles* species using female external morphology and employing traditionally used and new characters.

**Methods:**

Morphological characters of the females of South American species of the genus *Anopheles* were examined and employed to construct a species/group identification key. Photographs of key characters were obtained using a digital Canon Eos T3i, attached to a microscope. The program Helicon Focus was used to build single in-focus images by stacking multiple images of the same structure.

**Results:**

A morphological identification key to the adult females of species of the genus *Anopheles* described in South America is presented. Definitions and illustrations of the key characters are provided to facilitate use of key.

**Conclusions:**

Identification of species of the genus *Anopheles* based on female morphology is challenging because some key characters can be variable and overlapping among species. In addition, the majority of key characters are linked to color and shape of scales, their distribution on the head, scutum, abdomen, maxillary palpi, labium and legs, and pattern of pale and dark scales on dorsal and ventral surfaces of the wing veins. Thus, it is understandable that a specimen needs to be in good condition to be accurately identified. Morphologically similar species, such as those of the Konderi, Oswaldoi, Nuneztovari, Benarrochi and Albitarsis Complexes, and the Triannulatus and Strodei Groups, among others, cannot be accurately identified using characters included in the key. Further investigation will be required to exploit morphological characteristics for identification of members of those complexes, with formal description of new species.
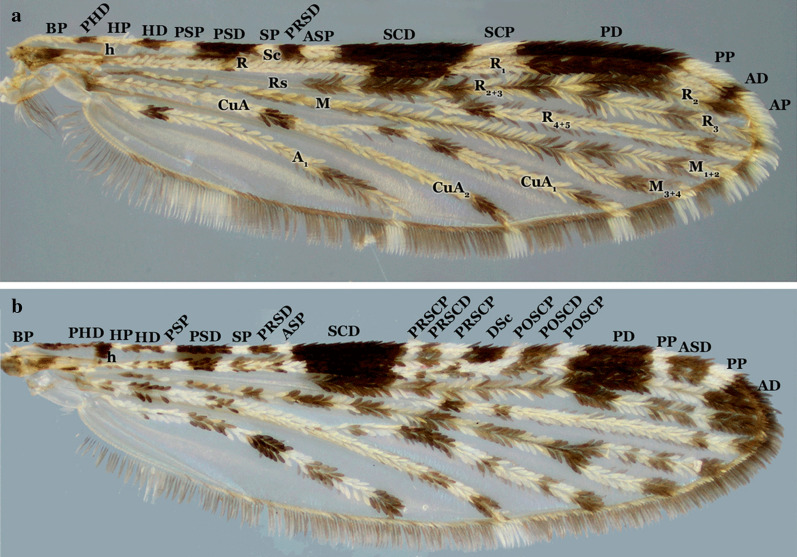

## Background

General introductory comments, distributions and species authors and publication dates are given in Part I [1] of this series of four articles. Keys to fourth-instar larvae and male genitalia are in Parts II [2] and III [3], respectively. Despite many recent studies have focused on the importance of DNA sequences for uncovering species complexes [4–13], the identification of *Anopheles* species is primarily based on morphological characters of female, male, and fourth-instar larvae [1]. This paper provides an illustrated dichotomous morphological key for the identification of females of *Anopheles* species of South America.

## Methods

The primary types (holotypes and paratypes) and other field-collected specimens deposited in the Coleção Entomológica de Referência, Faculdade de Saúde Pública, Universidade de São Paulo, São Paulo, Brazil (FSP-USP), Museo de Entomología, Universidad del Valle, Santiago de Cali, Colombia (MUSENUV) and the US National Mosquito Collection, Smithsonian Institution, Washington, DC, USA (USNMC) were selected and morphologically studied to discover additional characters to be used in the female key [1]. In addition, original descriptions, keys, summaries, and revisions from the published literature were examined. Photomicrographs of relevant characters for the female key were taken using a digital Canon Eos T3i (Canon, USA), attached to a stereomicroscope, using the program Helicon Focus software (https://www.heliconsoft.com/heliconsoft-products/helicon-focus/), which was used to build single in-focus images by stacking multiple images of the same structure. Photomicrographs were further processed in Adobe Photoshop (https://www.photoshop.com/en) to embed names and labels. Table 1 in Sallum et al. [1] shows the traditional classification of the genus *Anopheles*. The female key was modified from Forattini [14], Wilkerson & Strickman [15], and Harrison et al. [16] with further characters proposed herein.

## Results and discussion

Identification of species of the genus *Anopheles* based on female morphology can, for various reasons, be inaccurate. Morphological similarities and overlapping characters are common in species of the genus *Anopheles* and will increase with further taxonomic studies using molecular tools to address identification, phylogeny and establish species complexes. In addition, increased sampling in remote and poorly sampled regions of South America will propitiate discovery of new species and improvement in the taxonomic knowledge and nomenclature of the group as well. The newly proposed identification key compiled morphological information for identification of females, however, ideally characters of the male genitalia, fourth-instar larvae, and scanning electron microscope of the eggs should be examined to increase accuracy. Employment of this key to identify both unknown species and those already defined by molecular approach should be considered with caution. Likely, a specimen that may belong to a species that was not formally named will be identified to a morphologically similar species. Thus, when facing morphological variations, further investigations will be necessary to verify if those observed differences can indicate an unknown species. It is highly recommended to examine all life stages to reach an accurate species identification using morphology.

### Morphological features

**Fig. 1:**
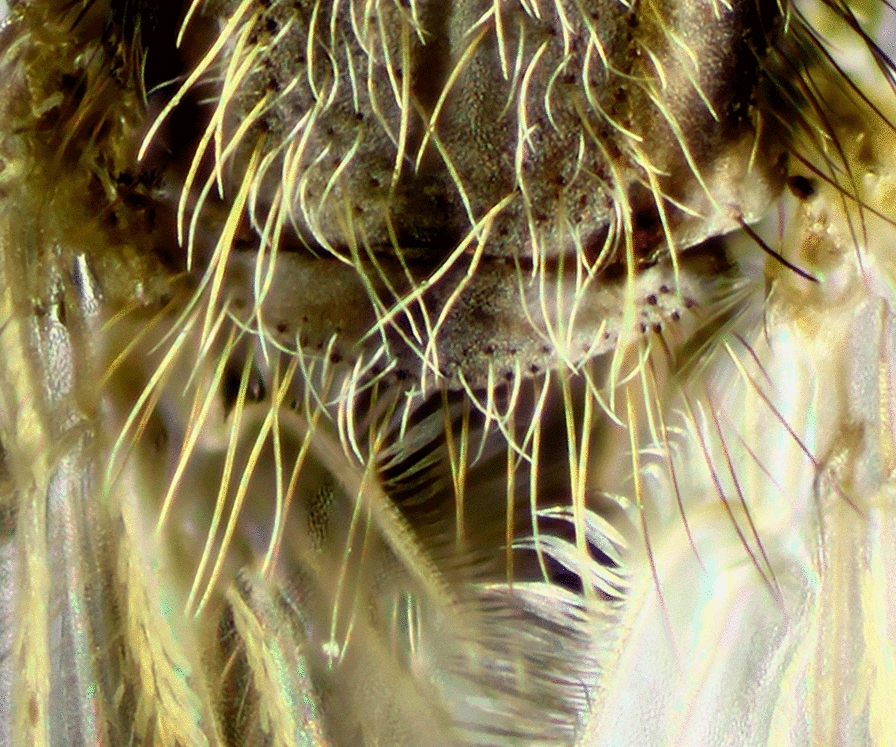
Uni-lobed scutellum of an adult of *An*. (*Ano*.) *pseudopunctipennis* Theobald, 1901

**Fig. 2:**
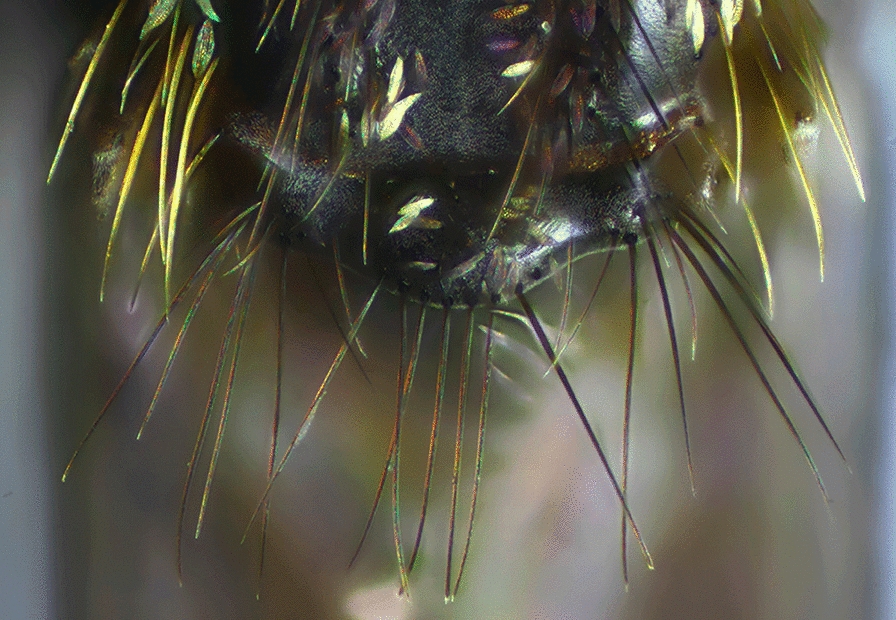
Tri-lobed scutellum of an adult of the genus *Chagasia* Cruz, 1906

The terminology of Harbach & Knight [17, 18] is followed in the key below. Valid species of the genus *Anopheles* of the subgenera *Anopheles*, *Kerteszia*, *Lophopodomyia*, and *Stethomyia* found in South America are provided in Table 1 in Sallum et al. [1]. In addition to the morphological traits that identify members of the Culicidae Meigen, 1818, most females of the subfamily Anophelinae Grassi, 1900 differ from those of the subfamily Culicinae Meigen, 1818 by having the maxillary palpi as long as the proboscis. In the Anophelinae, the majority of the species of the genera *Anopheles* Meigen, 1818 and *Bironella* Theobald, 1905 have the posterior margin of the scutellum rounded, not developed with median and lateral lobes. Consequently, the scutellar setae are uniformly distributed along the posterior border (Fig. 1). However, it is noteworthy that some species of the subgenera *Anopheles* and *Cellia* Theobald, 1902 exhibit a shallow subdivision into three lobes, but the distinction between the median and lateral lobes is not as evident as in species of the genus *Chagasia* Cruz, 1906 (Fig. 2).

#### Head

**Fig. 3:**
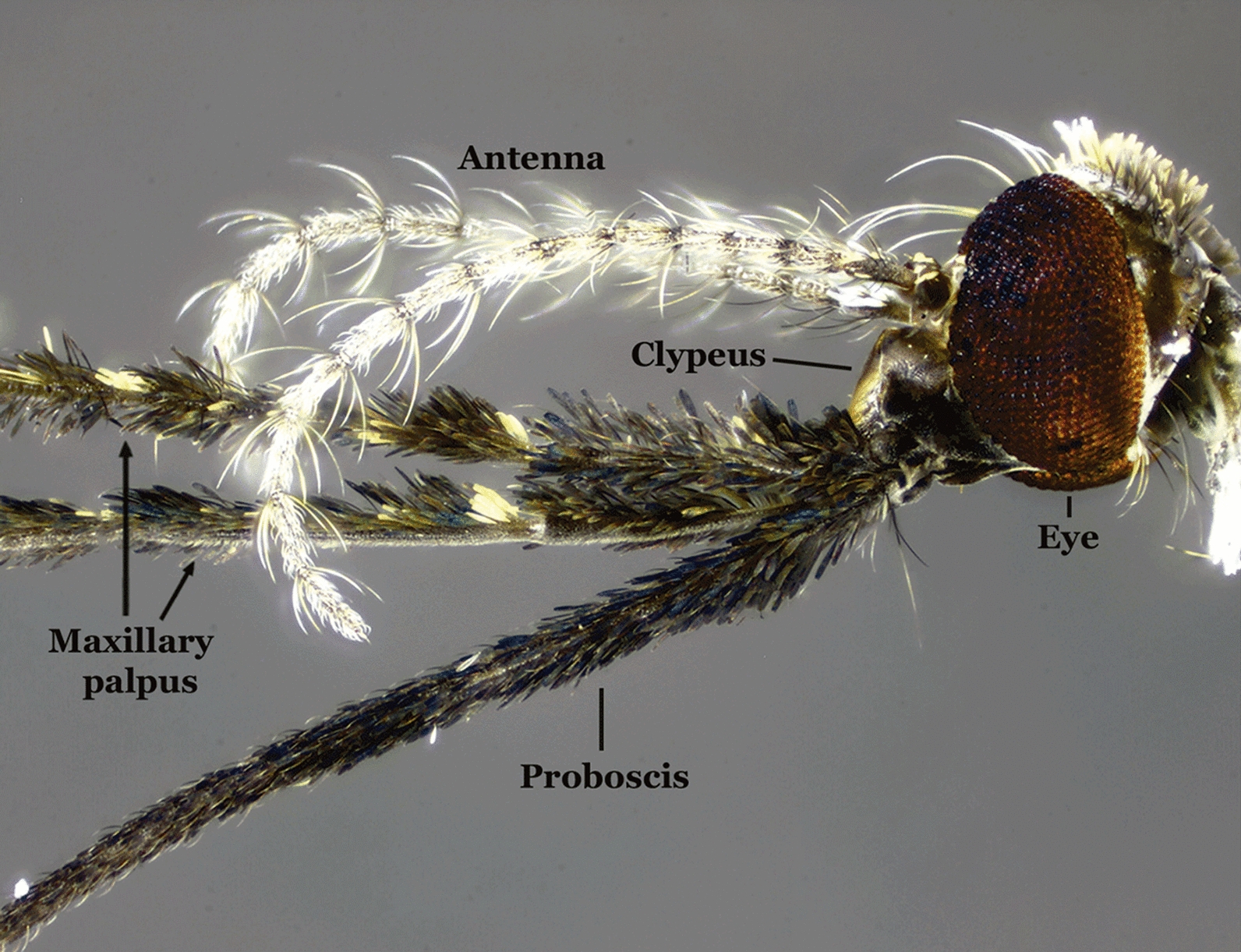
Head of a female of *An.* (*Ano*.) *calderoni* Wilkerson, 1991

*Anopheles*, like all other mosquitoes, have the antenna made up of 13 elongate flagellomeres. Each flagellomere possesses short setae dispersed around it and a number of longer, stronger setae arising apically (Fig. 3). In the males, the antenna possesses a higher concentration of longer and stronger setae disposed apically that form the flagellar whorl. The maxillary palpus of the females and males is made up of five palpomeres (Fig. 3). Palpomere 1 (MPlp_1_) is the shortest, arising laterally to the clypeus. Palpomere 5 (MPlp_5_) is longer than palpomere 1 but shorter than palpomeres 2, 3 and 4 (MPlp_2-4_), which are elongate. Scales covering the maxillary palpus vary in color from silvery white to cream to yellowish to dark brown and black. The pattern of distribution of pale and dark scales on the maxillary palpus can help identify some species of the genus *Anopheles*.

#### Thorax

**Fig. 4:**
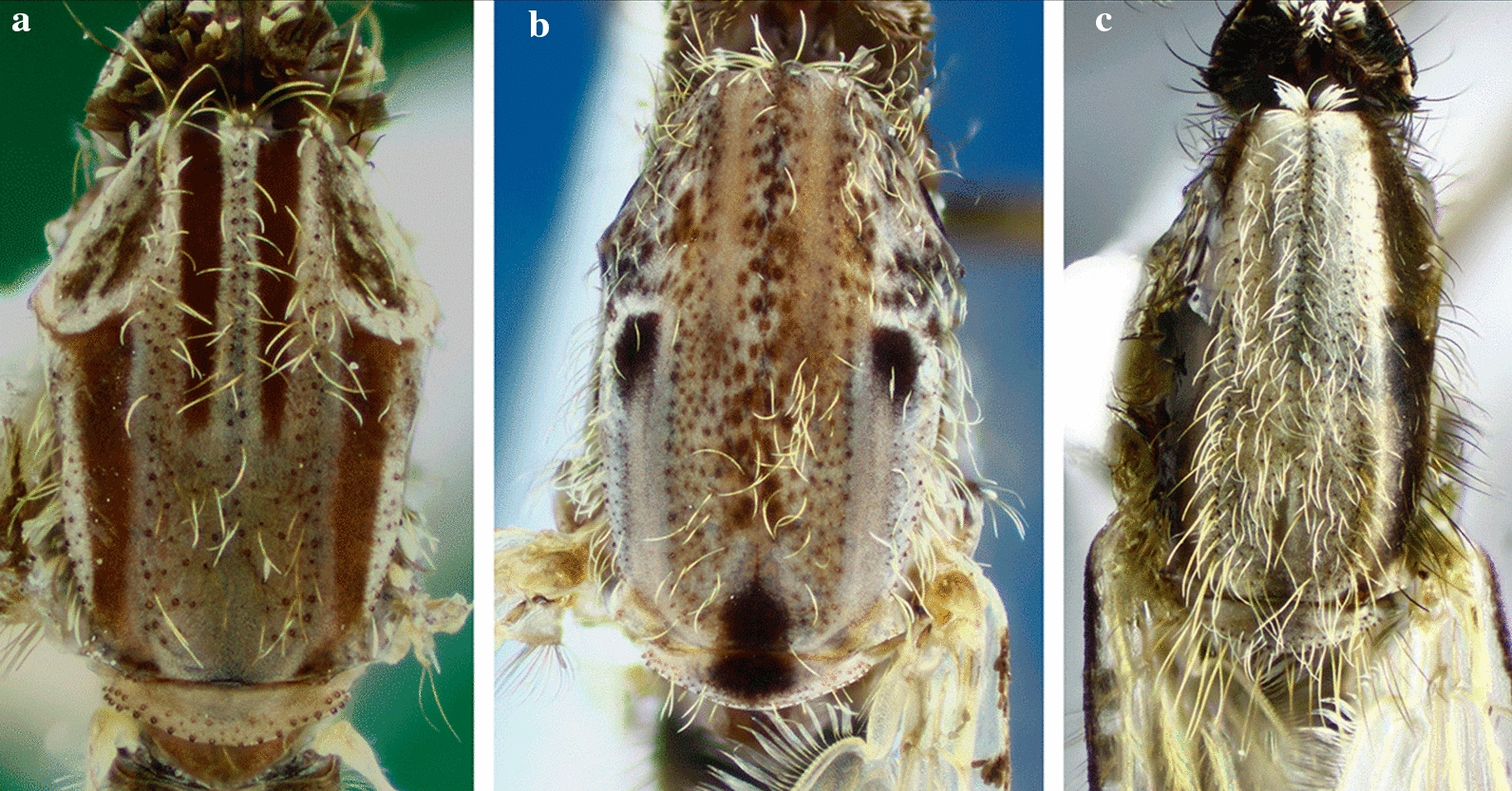
Thoraces of *Anopheles* spp., dorsal aspects. **a** *An*. (*Ker*.) *pholidotus* Zavortink, 1973. **b** *An.* (*Ano*.) *calderoni*. **c** *An.* (*Ano*.) *pseudopunctipennis*

**Fig. 5:**
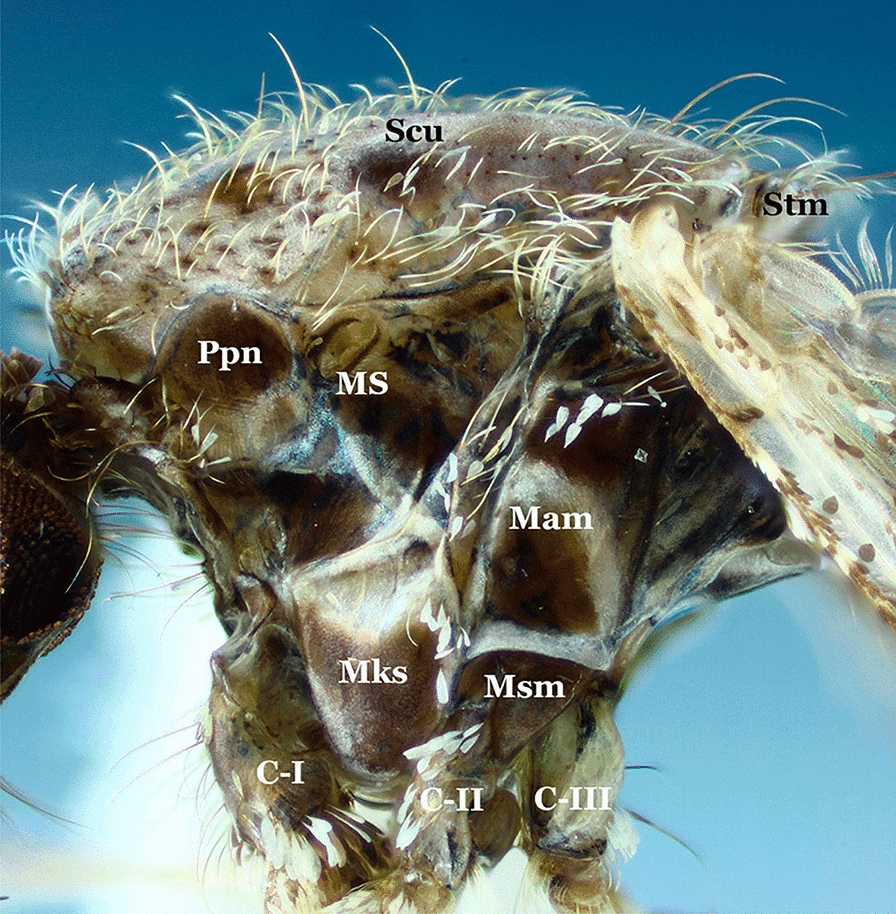
Thorax of *An*. (*Ano*.) *calderoni*, lateral aspect. *Abbreviations*: C-I, forecoxa; C-II, midcoxa; C-III, hindcoxa; Mam, mesepimeron; Mks, mesokatepisternum; MS, mesothoracic spiracle; Msm, mesomeron; Ppn, postpronotum; Scu, scutum; Stm, scutellum

**Fig. 6:**
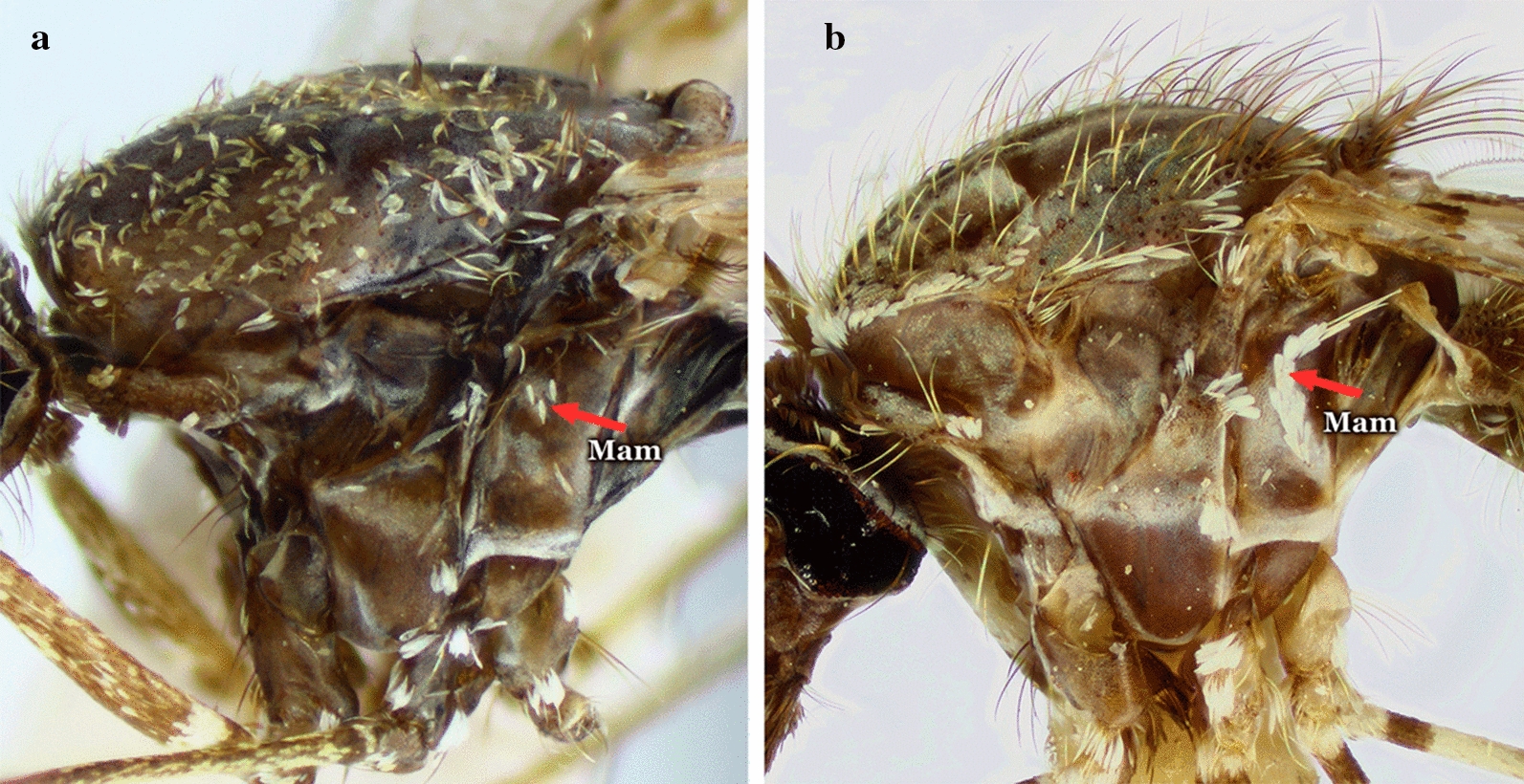
Thoraces of *Anopheles*, lateral aspects. **a** *An.* (*Nys*.) *darlingi* Root, 1926. **b** *An. pholidotus*

The thorax of the majority of the species of the genus *Anopheles* is elongate and as in all mosquitoes is represented mostly by the mesonotum (Fig. 4). The color of the scutal integument varies from blackish to brownish to grayish and exhibits patterns of color and scale distributions that can be employed for identification of species, species groups and subgenera. Scales can be absent or present. When present, scales are usually sparse and dispersed on some areas of the thoracic pleura (Fig. 5). The patterns of distribution of the scales on the mesokatepisternum and mesepimeron are frequently used to identify species of the subgenus *Kerteszia* Theobald, 1905 (Fig. 6).

#### Legs

**Fig. 7:**
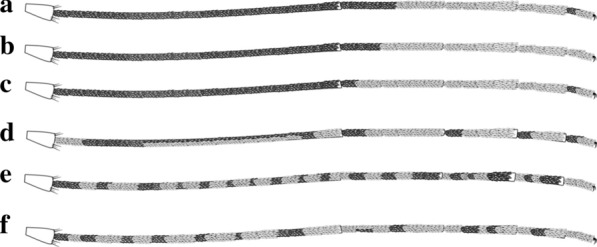
Hindtarsomeres of *Anopheles* spp. females. **a** *An.* (*Nys*.) *albimanus* Wiedemann, 1820. **b** *An.* (*Nys*.) *darlingi.* **c** *An.* (*Nys.*) *nuneztovari* Gabaldon, 1940. **d** *An.* (*Ker.*) *neivai* Howard, Dyar & Knab, 1913. **e** *An.* (*Ano*.) *malefactor* Dyar & Knab, 1907. **f** *An.* (*Ano.*) *costai* da Fonseca & da Silva Ramos, 1940

The legs of anophelines are predominantly dark but can have pale and dark scales in defined patterns or distributed without a characteristic pattern in the form of speckling. Some species have a defined pattern of scales, but there is also intraspecific and intra-individual variability. In other species, the legs are mostly dark-scaled, with pale scales forming rings and bands of variable size and distribution. On the hindlegs, the majority of species of the Arribalzagia Series of the subgenus *Anopheles*, as well as *Nyssorhynchus* Blanchard, 1902 and *Kerteszia*, have well-defined patterns of pale and dark scales that are often used for species identification. In species of the subgenus *Nyssorhynchus*, hindtarsomeres 2–5 are dark-scaled but show distinct patterns of pale scales that are employed for species identification (Fig. 7).

#### Wings

**Fig. 8:**
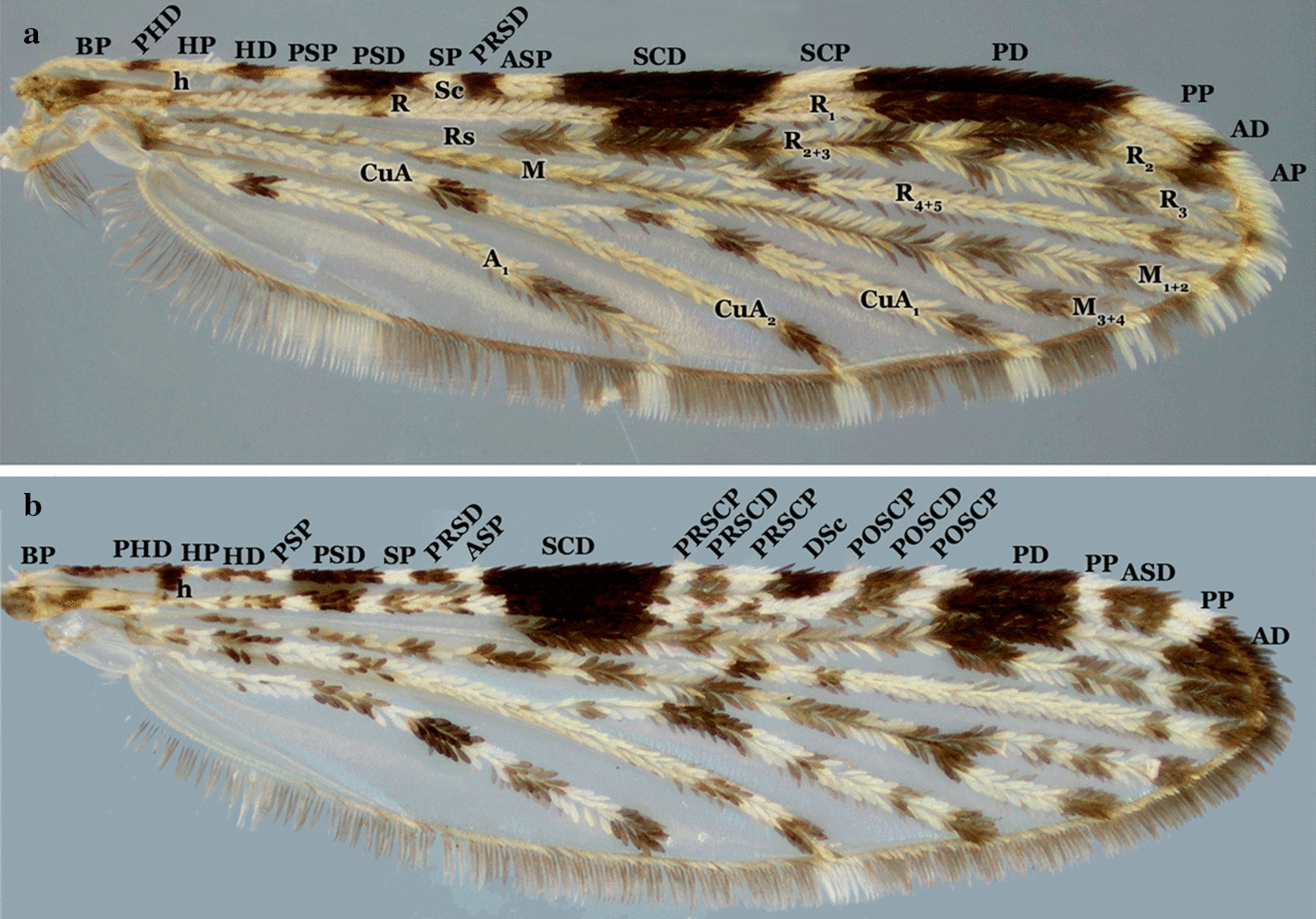
Nomenclature of wing veins and of pale and dark spots on the dorsal surface of *Anopheles* spp. wings. **a** *An. triannulatus*. *Abbreviations*: BP, basal plate; PHD, prehumeral dark; HP, humeral pale; HD, humeral dark; PSP, presector pale; PSD, presector dark; SP, sector pale; PRSD, proximal sector dark; ASP, accessory sector pale; SCD, subcostal dark; DSD, distal sector dark (when the ASP is missing, the composite dark spot is termed the SD, sector dark); SCP, subcostal pale; PD, preapical dark; PP, preapical pale; AD, apical dark; AP, apical pale. **b** *An. neomaculipalpus* Curry, 1931. Dark and pale spot names and abbreviations follow [19]. Spots are listed from left to right; those shown in panel **a** are followed by additional spots shown in panel **b**. Additional spots present in species of the Arribalzagia Series; subcostal vein ends in a AD, dark spot, SCD, subcostal dark in the middle of subcostal area. Spots basal to SCD are termed PRSCP, presubcostal pale and PRSCD, dark spots and those distal to it are the POSCP, postsubcostal pale and POSCD, dark spots. Also, in species of the series, the PP, preapical pale is interrupted by an ASD, accessory preapical dark

**Fig. 9:**
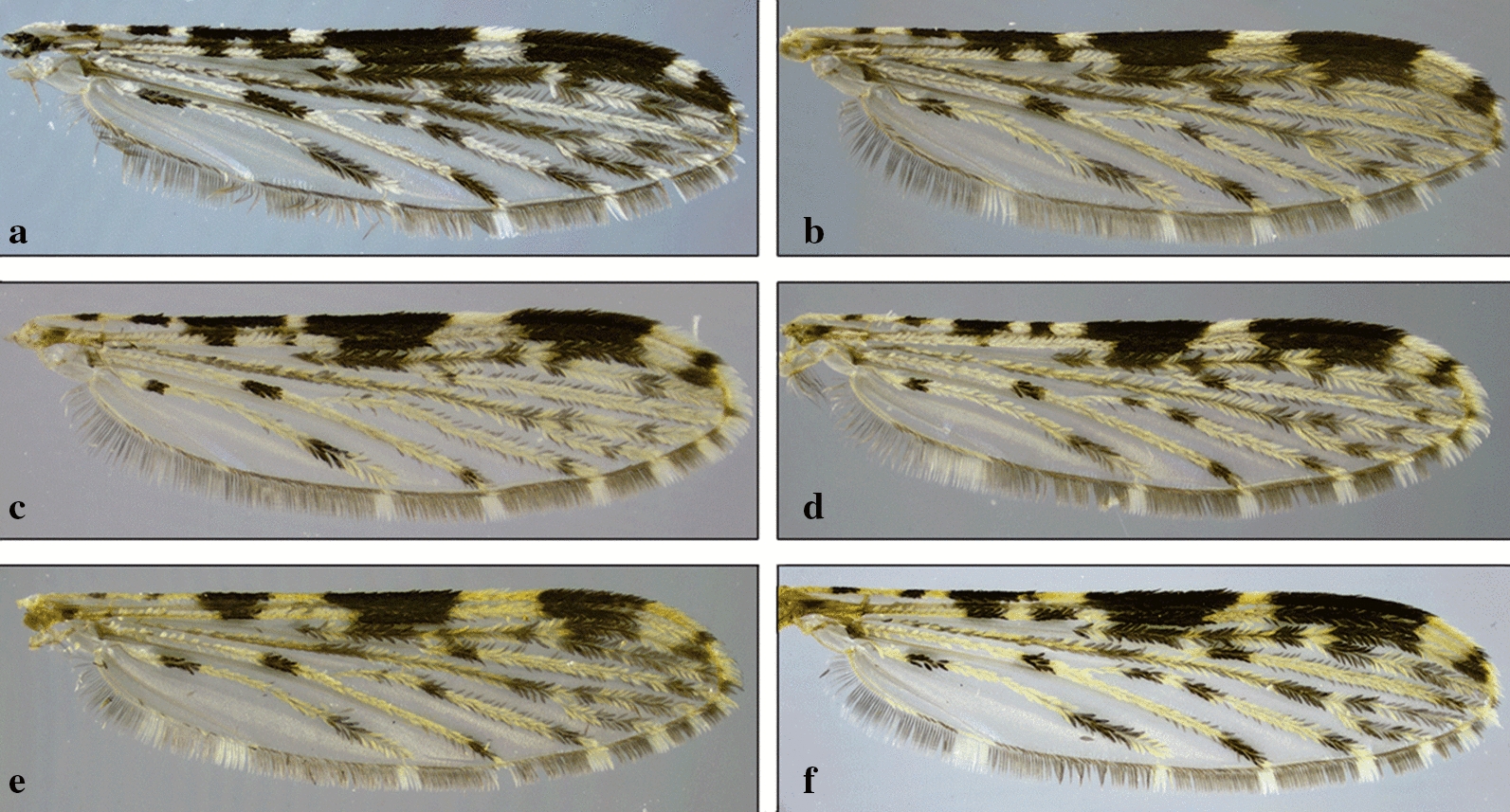
Pale and dark wing spots in species of *Anopheles* (*Nyssorhynchus*). **a** *An*. *braziliensis* (Chagas, 1907). **b** *An*. *albitarsis* Lynch Arribálzaga, 1878. **c** *An*. *strodei* Root, 1926. **d** *An*. *triannulatus* (Neiva & Pinto, 1922). **e** *An*. *nuneztovari*. **f** *An*. *albimanus*

**Fig. 10:**
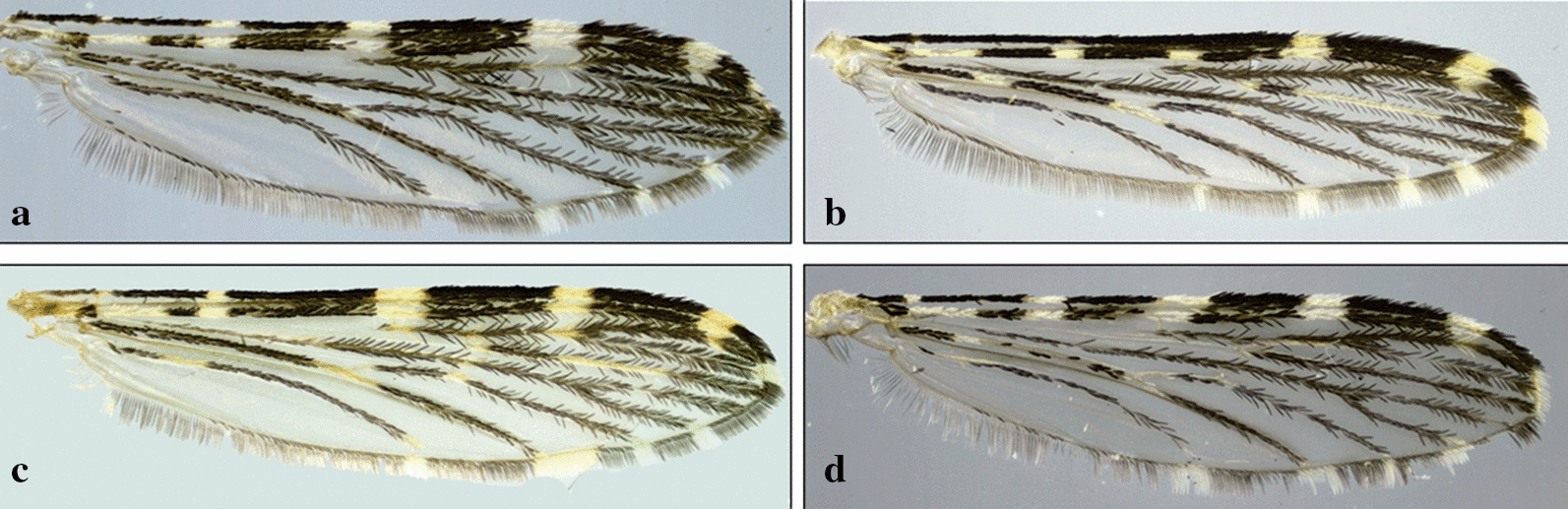
Wings of species of *Anopheles* (*Kerteszia*). **a** *An*. *pholidotus*. **b** *An*. *homunculus* Komp, 1937. **c** *An*. *gonzalezrinconesi* Cova Garcia, Pulido F. & Escalante de Ugueto, 1977. **d** *An*. *neivai*

**Fig. 11:**
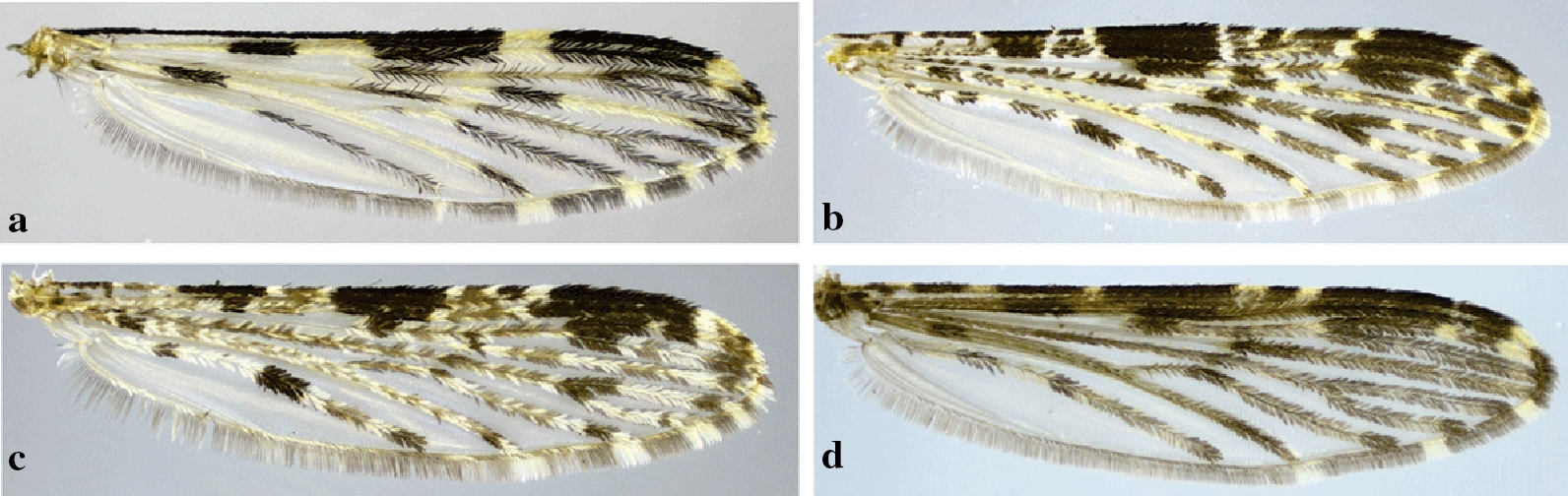
Wings of species of *Anopheles* (*Anopheles*). **a** *An*. *pseudopunctipennis*. **b** *An*. *calderoni*. **c** *An*. *peryassui* Dyar & Knab, 1908. **d** *An*. *mattogrossensis* Lutz & Neiva, 1911

Independent of the shading or dark patterns that are sometimes seen on the wing membrane, the coloration of the scales that cover most of the wing veins is what defines the color of the wings. The scales vary from dark to pale, making the wings appear completely dark or with pale and dark areas that form patterns that are species-specific or group specific (Figs. 8, 9, 10, 11). This is usually evident on the longitudinal veins. The nomenclature adopted in the identification key is that proposed by Wilkerson & Peyton [19]. The wing spots are named with reference to the pale and dark spots observed in *An*. (*Cellia*) *kochi* Dönitz, 1901 and *An*. (*Anopheles*) of the Arribalzagia Series (see Fig. 8a, b for names and abbreviations of wing spots).

#### Abdomen

**Fig. 12:**
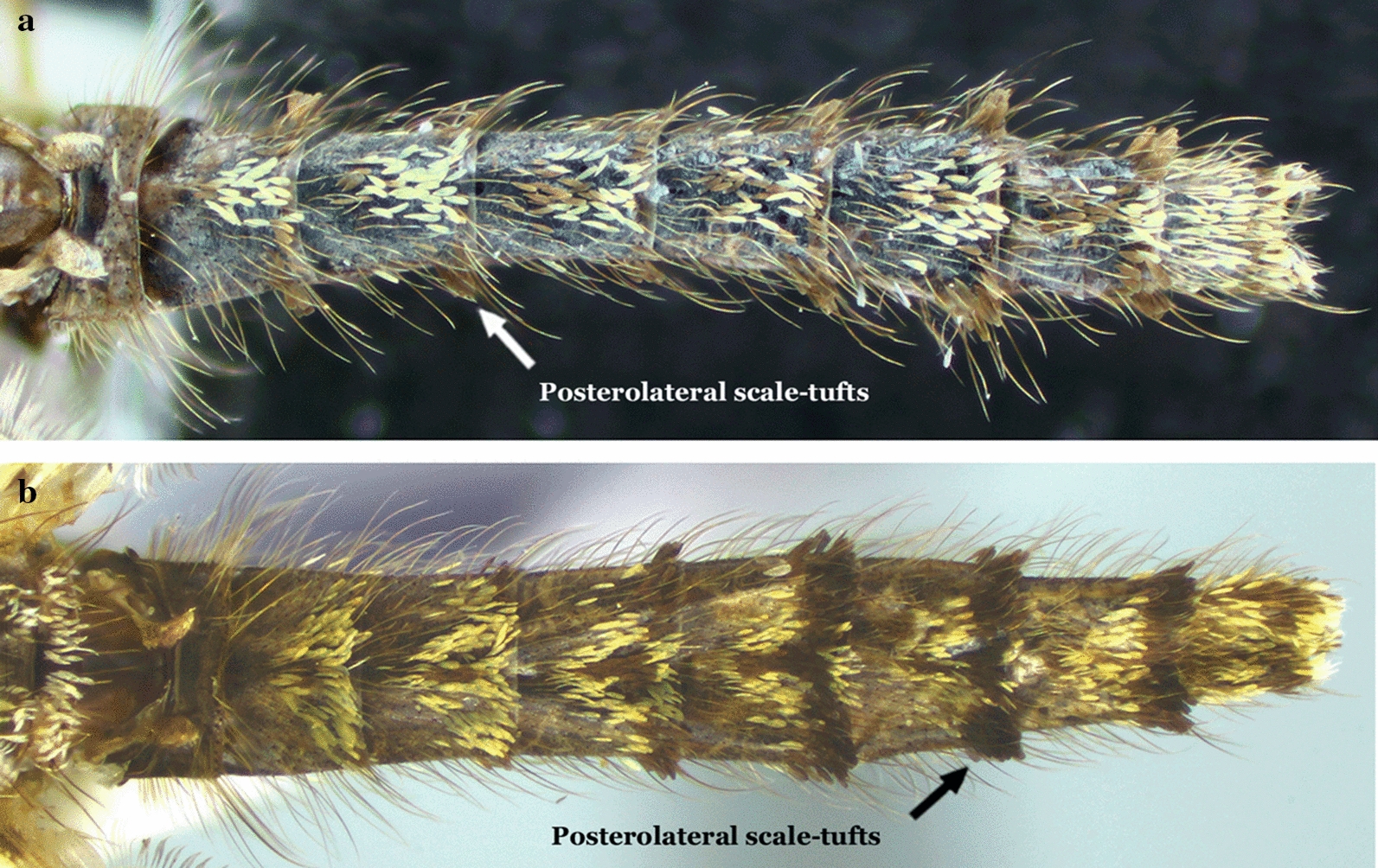
Abdomens of *Anopheles* spp., dorsal view. **a** *An*. (*Nys*.) *darlingi*. **b** *An*. (*Nys*.) *albimanus*

**Fig. 13:**
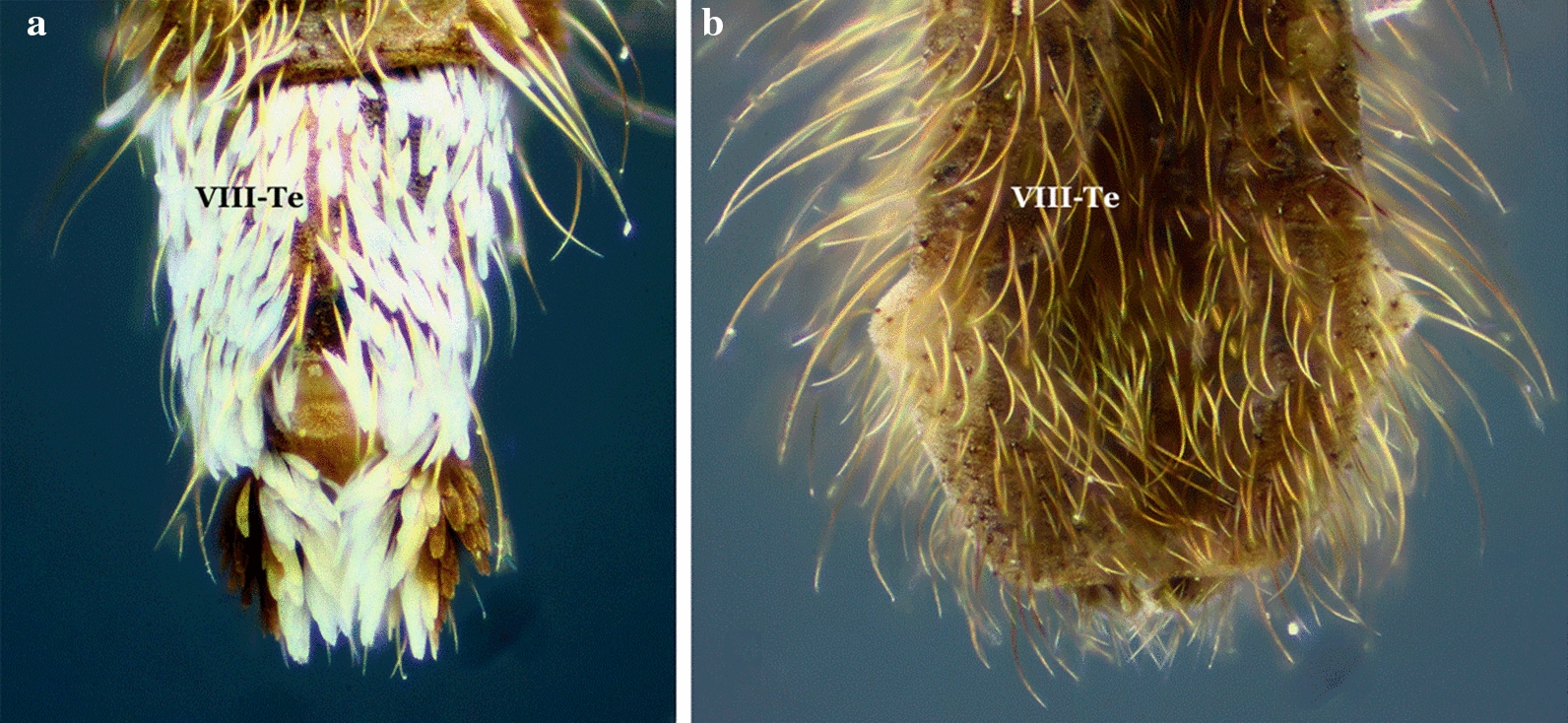
End of abdomen; tergum VIII of *Anopheles* spp., dorsal view. **a** *An*. (*Ano*.) *peryassui*. **b** *An*. (*Ano*.) *mattogrossensis*

**Fig. 14:**
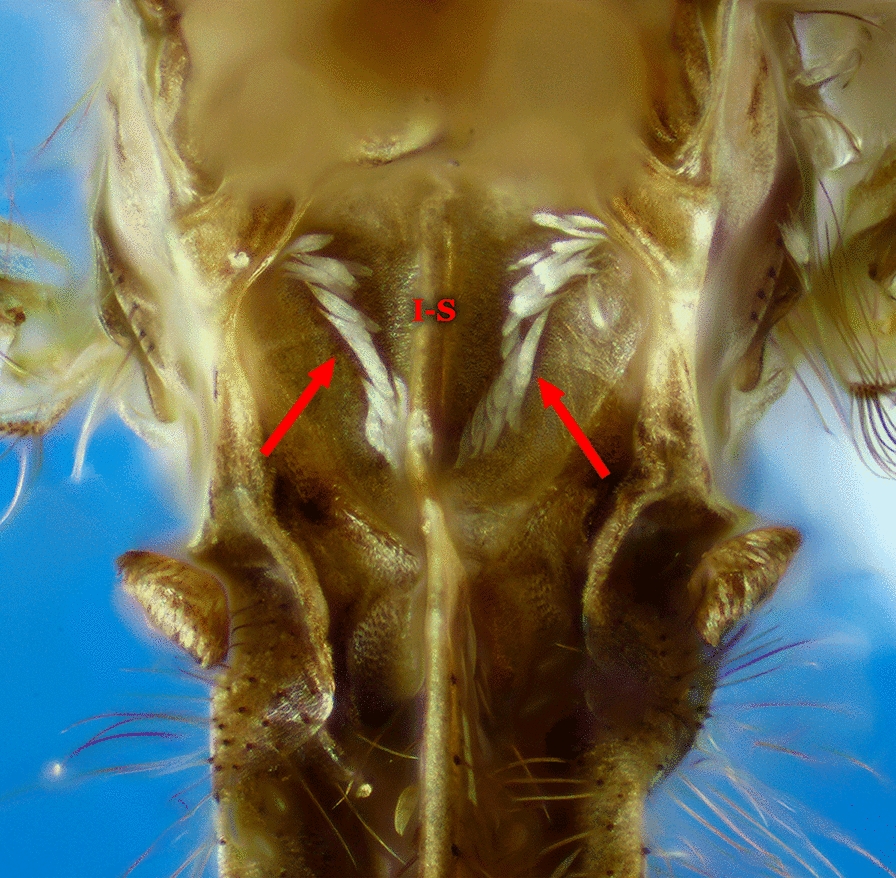
Abdominal sternum I, *An*. (*Nys*.) *albitarsis*

Females of the genus *Anopheles* possess a variable pattern of scales, ranging from a dense covering (Fig. 12), i.e. *Anopheles pharoensis* Theobald, 1901 (an African species), to scales grouped in patches that are more evident on the dorsal portions of the segments, to almost entirely bare. The absence of scales on the abdominal segments is variable and is observed in species of diverse subgenera of the genus *Anopheles*. However, the abdomen is always covered with setae of variable development. The majority of the species of the subgenus *Nyssorhynchus* and some species of the subgenus *Anopheles* possess patches of scales grouped laterally at the posterior end of segments II-VII or III-VII or IV-VII. These patches of scales are called posterolateral scale-tufts (Fig. 11). In other species, scales are either absent or present only on segments VII and VIII and the cerci (Fig. 13). Abdominal sternum I is small and closely associated with the metathorax. Consequently, it is usually not easy to examine characteristics of sternum I when the specimen is dry-pinned, and the abdomen droops. Traits of sternum I are more easily seen if the individual is examined from a posterior view. In some species of the subgenus *Nyssorhynchus*, sternum I possesses sparse scales, or the scales are arranged in a longitudinal line (Fig. 14).

The morphological key provides diagnostic characters in couplets for identifications of specimens of species of the genus *Anopheles* of South America. The subgenus is marked in the couplet that is linked to the species of that taxonomic group. Characters employed in the key can be seen with a light stereomicroscope. Wing spots and scale color are critical and need to be examined with sufficient light that does not distort the color, ideally with a day light filter, and a microscope scale to calculate length ratios of some characters, such as fore- and hindtarsomeres, and dark and pale wing scale spots.

### Key for the identification of species of the genus *Anopheles* of South America based on morphological characters of the adult females

- Integument of scutum with a median longitudinal silvery stripe, dark laterally; head mostly without scales, except for some erect scales on vertex; wing veins and legs covered with dark scales (subgenus *Stethomyia* Theobald, 1902)……2
- Scutum otherwise; head with numerous erect scales on vertex and occiput; wing veins variably covered with pale and dark scales……3
- Setae and scales of the frontal tuft long, extending beyond antennal pedicels; lateral margin of the scutum with silvery stripe, as distinct and developed as the median stripe……*An. nimbus, An. thomasi* & *An. acanthotorynus*
- Setae and scales of frontal tuft short, not extending beyond antennal pedicels; lateral margin of scutum, if with a silvery stripe, not as developed as median stripe……*An. kompi* & *An. canorii*
- Integument of scutum with 4 distinct, longitudinal, silvery pruinose stripes intermixed with dark pruinose longitudinal stripes (subgenus *Kerteszia*)……4
- Integument of scutum variable, not as above……13
- Mesepimeron with a vertical C-shaped scale-patch (Fig. 6b) that begins at upper mesepimeral setae and continues ventrally……5
- Mesepimeron with 1 or 2 small white scale-patches……6
- Proboscis, pedicel and palpomere 1 (MPlp_1_) white-scaled; hindtarsomeres 1 and 2 (Ta-III_1,2_) without apical, pale bands (in dorsal view)……*An. lepidotus*
- Proboscis, pedicel and palpomere 1 (MPlp_1_) without white scales; hindtarsomeres 1 and 2 (Ta-III_1,2_) with apical, pale bands (in dorsal view) (Figs. 4a, 6b, 10a)……*An. pholidotus*
- Mesepimeron with a small patch of scales inserted near the upper mesepimeral setae ………7
- Mesepimeron with 2 small patches of scales (upper and median)……10
- Abdominal terga II-VII (II-VII-Te) covered with numerous dark decumbent scales; abdominal sterna with, sparse white scales (Fig. 10c)……*An. boliviensis*, *An. gonzalezrinconesi* & *An. rollai*
- Abdominal terga and sterna without scales, occasionally with a few scales on segments VII and VIII and cerci……8
- Hindtarsomere 5 (Ta-III_5_) entirely white-scaled; wing without pale apical fringe spot……*An. bambusicolus*
- Hindtarsomere 5 (Ta-III_5_) dark proximally, distal 0.35–0.60 pale; wing with large pale apical fringe spot, rarely this spot divided into 2 small pale spots……9
- Scutum with pale scales on acrostichal area, scales extending from anterior promontory nearly to prescutellar setae; hindtarsomeres 2–4 (Ta-III_2−4_) each with narrow pale band on distal 0.15–0.5……*An. auyantepuiensis*
- Scutum without pale scales on acrostichal area; hindtarsomeres 2–4 (Ta-III2−4) each with broad white band on distal 0.5–0.7 (Figs. 7d, 10d)……*An. neivai* (*s.l.*)
- Hindtarsomeres 2–4 (Ta-III_2−4_) each with narrow apical pale stripe 0.3 or less length of tarsomeres; hindtarsomere 5 (Ta-III_5_) usually entirely dark-scaled, infrequently pale-scaled apically……*An*. *bellator*
- Hindtarsomeres 2–5 (Ta-III_2−5_) each with a broad, apical pale band, extending from 0.4 to 0.7……11
- Scutum with anterior 0.3–0.4 of acrostichal and dorsocentral areas and middle of scutellum with a few white scales; vein M entirely or mostly white-scaled basal to level of bifurcation of vein CuA……*An*. *laneanus*
- Scutum without pale scales on acrostichal and dorsocentral areas and scutellum; vein M with dark scales basal to level of bifurcation of vein CuA……12
- Palpomeres 3 and 4 (MPlp_3,4_) covered predominantly by decumbent scales, sometimes those at base of palpomere 3 (MPlp_3_) slightly erect……*An*. *cruzii*
- Palpomere 3 (MPlp_3_) covered with slightly erect scales, palpomere 4 (MPlp_4_) with slightly erect to decumbent scales (Fig. 10b)……*An*. *homunculus*
- Femora and tibiae unicolorous or variously marked, if speckled with pale and dark spots, dark spots are few and small; vein C with a single small to large pale spot (subcostal pale, SCP) in vicinity of junction with subcostal vein (Sc), or vein C entirely dark at junction with subcostal vein (Sc); sector pale spot (SP), if present, not interrupted by the accessory sector dark spot (ASD)……14
- Femora and tibiae speckled with numerous large pale spots; vein C with a small to large dark spot (subcostal dark (SCD)) at junction with subcostal vein (Sc), dark spot bordered on each side by one or more precoacal (PRSCP, PRSCD) and postsubcostal (POSCP, POSCD) pale and dark spots; sector pale spot (SP) interrupted by an accessory sector dark (ASD) spot……18
- Hindfemur (Fe-III) with a distinct apical patch of erect dark scales……*An*. *squamifemur*
- Hindfemur (Fe-III) without an apical patch of erect dark scales……15
- Hindtarsomeres (Ta-III_1−5_) predominantly dark-scaled, without conspicuous pale stripes, at most with small basal spots or very narrow stripes of pale scales on some tarsomeres……16
- Hindtarsomeres (Ta-III_1−5_) each with conspicuous pale apical stripe, or some posterior tarsomeres with conspicuous pale apical stripe and others completely white……44
- Wings almost totally dark-scaled; pale spots, when present, limited in number and small……17
- Coloration of wing scales variable, spots pale or dark but with more and variable in length pale spots……38
- Wing fringe with distinct pale spots at apices of veins R_2_, R_3_ and R_4+5_; known distribution Central America……*An*. *eiseni eiseni*
- Wing fringe with distinct pale spots at apices of veins R_3_ and R_4+5_; known distribution South America……*An*. *eiseni geometricus*
- Abdominal segments without erect or semi-erect posterolateral scale-tufts……19
- Abdominal segments with erect or semi-erect posterolateral scale-tufts……21
- Tergum VIII (VIII-Te) densely covered with white or grayish scales, sometimes with dark scales basally and pale scales apically; scutum and scutellum with 3 distinct dark spots accentuated by silvery pruinosity, 2 spots situated laterally, posterior to wing, and 1 situated in prescutellar area, reaching anterior part of median lobe of scutellum (Figs. 11a, 13a)……*An*. *peryassui*
- Tergum VIII (VIII-Te) without white scales; integument of scutum and scutellum homogeneously dark without pattern or pruinose patches of dark spots……20
- Subcostal area (SCA) on vein C with 1 dark and 2 pale spots; subcostal area on veins R_1_ and R_2+3_ predominantly pale-scaled; preapical dark spot (PD) fused with the accessory preapical dark (APD); preapical pale spot (PP) present at apex of vein R_1_……*An*. *vestitipennis*
- Subcostal area on vein C with 1 dark and 2 pale spots; subcostal area (SCA) on veins R_1_ and R_2+3_ predominantly dark-scaled; preapical dark area (PD) separated from accessory preapical dark (APD), preapical pale area (PP) with 2 pale spots, interrupted by accessory preapical dark (APD) (Figs. 11d, 13b)……*An*. *mattogrossensis*
- Hindtarsomeres 2–4 (Ta-III_2−4_) mostly dark-scaled, with only apical pale rings and some basal pale scales at articulations……22
- Hindtarsomeres 2–4 (Ta-III_2−4_) with more pale-scaled areas than above……24
- Hindtarsomere 1 (Ta-III_1_) with various pale spots……*An*. *minor*
- Hindtarsomere 1 (Ta-III_1_), dark with an apical pale ring……23
- Wing vein R_4+5_ with a mixture of pale and dark spots; subcostal dark spot (SD) large, extending anteriorly from union of subcosta (Sc) with costa (C); pre- and postsubcostal dark spots well defined……*An*. *shannoni*
- Wing vein R_4+5_ with 3 distinct dark spots; subcostal dark spot (SD) small, confined to union of subcostal vein (Sc) with costa; pre- and postsubcostal (PRSCP, POSCP) dark spots not well defined……*An*. *guarao*
- Hindtarsomere 5 (Ta-III_5_) entirely pale……25
- Hindtarsomere 5 (Ta-III_5_) with a dark spot……29
- Upper mesepimeral scales absent……*An*. *punctimacula* (in part)
- Upper mesepimeral scales present……26
- Sternum I (I-S) with a small patch or line of scales (Fig. 7f)……*An*. *mediopunctatus*, *An*. *costai* & *An*. *forattinii*
- Sternum I (I-S) without scales……27
- Hindtarsomere 4 (Ta-III_4_) with 3 pale spots of variable size, sometimes entirely pale; postsubcostal dark spot (POSCD) on costa (C) small, poorly defined……*An*. *fluminensis*
- Hindtarsomere 4 (Ta-III_4_) dark or with few pale scale-spots, never entirely pale; postsubcostal dark spot (POSCD) on costa (C) large, well-defined……28
- Wing with postsubcostal pale spot (POSCP) on costa (C) contiguous with corresponding pale spot on R_1_ (Fig. 7e)……*An*. *malefactor*
- Postsubcostal pale spot (POSCP) on costa (C) separated by dark scales from corresponding spot on R_1_ (Figs. 3, 4b, 5, 11b)……*An*. *calderoni*
- Wing with narrow scales basally, scale length ≥ 3 times width at widest point……30
- Wing with broad scales basally, scale length < 3 times width at widest point……32
- Wing with small preapical dark spot (PD), 0.06–0.12 length of wing; costa (C) with 2 primary dark spots (SD and PD), presector dark spot (PSD) reduced in size (Fig. 8)……*An*. *neomaculipalpus*
- Wing with preapical dark spot (PD) larger, 0.11–0.23 length of wing; costa (C) with 3 primary dark spots (SD, PD and PSD)……31
- Hindtarsomere 3 (Ta-III_3_) with a basal dark ring; midtarsomere 5 (Ta-II_5_), completely dark; vein R_1_ with the dark spot in subcostal area (SCA) interrupted by a pale spot in line with subcostal dark spot (SCD) on costa (C); accessory sector dark spot (ASD) on costa (C) does not clearly extending to vein R_1_……*An*. *anchietai*
- Hindtarsomere 3 (Ta-III_3_), with a basal pale ring; midtarsomere 5 (Ta-II_5_) dark basally and pale apically; vein R_1_ with dark spot in subcostal area (SCA) without a pale interruption; accessory sector dark spot (ASD) on costa (C) clearly extends to vein R_1_……*An*. *maculipes* & *An*. *pseudomaculipes*
- Wing vein CuA mostly dark-scaled……33
- Wing vein CuA mostly pale-scaled……36
- Wing vein 1A with distal half dark-scaled……*An*. *bustamentei*
- Wing vein 1A with pale and dark areas along entire length……34
- Scales on middle portion of anterior cubital vein (CuA) dark, scales decumbent and smaller than on other veins; anterior wing veins with 4 primary dark spots, apical dark spot (AD) as distinct as preapical dark (PSD), sector dark (SD) and preapical dark (PD) spots……*An*. *apicimacula* (in part)
- Scales on middle portion of anterior cubital vein (CuA) not as above, predominantly pale or with a mixture of pale and dark scales, usually not decumbent, about same size as scales on other veins; anterior wing veins with 3 or 4 primary spots, apical dark spot (AD) either distinct or indistinct……35
- Preapical dark spot (PD) on costa (C) shorter than sector dark spot (SD (DSD)……*An*. *medialis*
- Preapical dark spot (PD) on costa (C) about same length as sector dark spot (SD (DSD))……*An*. *calderoni*
- Wing vein R_4+5_ with 2 well-defined dark spots, one basal and the other apical; vein 1A with 3 or 4 dark spots……*An*. *rachoui*
- Wing vein R_4+5_ with 4 well-defined dark spots, 2 basal and 2 apical; vein 1A with 6 or more dark spots……37
- Postsubcostal pale spot (POSCP) on costa (C) not contiguous with corresponding spot on R_1_; costa (C) straight at union of subcostal vein (Sc)……*An*. *evandroi*
- Postsubcostal pale spot (POSCP) on costa (C) contiguous with corresponding spot on R_1_; costa (C) distinctly emarginated at union of subcostal vein (Sc)……*An*. *punctimacula*
- Hindtibia (Ti-III) with a large pale area on apical 0.25……39
- Hindtibia (Ti-III) without a large apical pale area, at most with a small spot or pale ring……41
- Vein 1A entirely dark-scaled……*An*. *tibiamaculatus*
- Vein 1A with pale- and dark-scaled areas……40
- Scutum covered with grayish or whitish pruinosity……*An*. *gilesi*
- Scutum covered with grayish pruinosity, forming a broad median longitudinal band, dark brown laterally……*An*. *pseudotibiamaculatus*
- Wing vein 1A entirely dark-scaled, or at most with a small median pale spot……*An*. *vargasi*
- Wing vein 1A pale- or dark-scaled, if dark-scaled then always with more than 1 pale spot……42
- Hindfemur (Fe-III) and hindtibia (Ti-III) uniformly dark, sometimes with small apical pale ring; costa (C) with 2 pale spots, one at the point of union with subcosta (SCP), the other on preapical area (PP); vein 1A with basal 0.5 dark-scaled, distal 0.5 pale-scaled, and with a small pale spot at apex (Figs. 4c, 11a)……*An*. *pseudopunctipennis*
- Hindfemur (Fe-III) and hindtibia (Ti-III) speckled with white and yellow spots; costa (C) with more than 2 pale spots; vein 1A with a variable pattern of pale and dark spots……43
- Wing vein R_4+5_ with 2 dark spots separated by an intermediate pale spot; vein 1A with 3 dark spots……*An*. *oiketorakras*
- Wing vein R_4+5_ with 1 distal dark spot; vein 1A with 2 dark spots……*An*. *gomezdelatorrei*
- Wing vein 1A with about 5 pale spots interspersed with 6 dark spots, all small; hindfemur (Fe-III) and hindtibia (Ti-III) dark, speckled with pale spots of variable size; hindtarsomeres 4 and 5 (Ta-III_4,5_), entirely white……*An*. *annulipalpis*
- Wing vein 1A with fewer pale and dark spots; hindfemur (Fe-III) and hindtibia (Ti-III) dark with sparse pale scales, not speckled; hindtarsomeres 4 and 5 (Ta-III_4,5_) white or white with dark rings at base or apex……45
- Abdominal terga (Te), at least segments II-VII (II-VII-Te), covered with scales……46
- Abdominal terga (Te) with or without sparse scales (Myzorhynchella Section)……59
- Hindtarsomere 5 (Ta-III_5_) with a basal dark spot……47
- Hindtarsomere 5 (Ta-III_5_) entirely white-scaled or with an apical dark ring……63
- Abdominal segment II without posterolateral scale-tufts; palpomere 4 (MPlp_4_) completely dark-scaled or with yellowish or golden-brown mediolateral scales, never white or cream-colored (Figs. 7a, 9f, 12b)……*An*. *albimanus*
- Abdominal segment II with posterolateral scale-tufts; palpomere 4 (MPlp_4_) differently marked, with at least some white or cream-colored mediolateral scales……48
- Anterior mesepimeron with a conspicuous white scale-patch; wing subcostal pale spot (SCP) reduced; prehumeral dark spot (PHD) extends to the humeral crossvein (h) (Figs. 8a, 9d)……*An*. *halophylus*, *An*. *triannulatus* & *An*. *triannulatus* C
- Anterior mesepimeron without a conspicuous white scale-patch, sometimes with 1 or 2 small pale scales; wing subcostal pale spot (SCP) variable, never reduced; prehumeral dark spot (PHD) variably developed……49
- Hindtarsomere 3 (Ta-III_3_) variable, sometimes with a basal dark band ≤ 0.4 length of tarsomere; prescutelar area covered with a distinct large dark spot……*An*. *rondoni*
- Hindtarsomere 3 (Ta-III_3_) completely pale; prescutelar area with a more-or-less distinct small spot, but never entirely covering prescutelar area……50
- Hindtarsomere 2 (Ta-III_2_) with a basal dark spot < 0.25 length of tarsomere……51
- Hindtarsomere 2 (Ta-III_2_) with a basal dark spot > 0.25 length of tarsomere……52
- Foretarsomere 4 (Ta-I_4_) entirely pale or, rarely with more than basal 0.30 dark; midtarsomere 4 (Ta-II_4_) with an apical pale stripe corresponding to 0.15–0.25 length of segment; foretarsomeres 3–5 (Ta-I_3−5_) mostly cream-colored, sometimes white, dark scales frequently present only on dorsobasal surface; foretarsomere 2 (Ta-I_2_) pale on apical 0.35–0.55; foretarsomere 3 (Ta-I_3_) pale on apical 0.70–0.86……*An*. *ininii*
- Foretarsomere 4 (Ta-I_4_) entirely dark, at least on basal third; midtarsomere 4 (Ta-II_4_) entirely dark; foretarsomeres 3–5 (Ta-I_3−5_) with pale scales nearly forming a complete ring on all tarsomeres, dark scales occasionally absent on ventral surface; foretarsomere 2 (Ta-I_2_) pale on apical 0.20–0.45; foretarsomere 3 (Ta-I_3_) pale on apical 0.50–0.85……*An*. *oswaldoi* & *An*. *konderi*
- Costa (C) with subcostal pale spot (SCP) > 0.5 length of sector dark spot (SD)……*An*. *rangeli*
- Costa (C) with subcostal pale spot (SCP) < 0.5 length of the sector dark spot (SD)……53
- Wing with vein M stem predominantly dark-scaled from apex to basal third, vein M_1_ with predominantly dark scales…………*An*. *bennarrochi*
- Wing with vein M stem predominantly pale-scaled……54
- Hindtarsomere 2 (Ta-III_2_) with dark spot extending beyond basal 0.5……*An*. *aquasalis* & *An*. *galvaoi*
- Hindtarsomere 2 (Ta-III_2_) with dark spot never extending beyond basal 0.5……55
- Humeral pale spot (HP) on costa (C) < 2.0 length of prehumeral dark spot (PHD)……56
- Humeral pale spot (HP) on costa (C) ≥ 2.0 length of prehumeral dark spot (PHD)……57
- Pale wing scales very pale to pale cream-colored; costa (C) with humeral pale spot (HP) 1.3–2.0 length of prehumeral dark spot (PHD)……*An*. *dunhami* & *An*. *trinkae*
- Pale wing scales, at least on anterior veins, yellowish to cream-colored; costa (C) with humeral pale spot (HP) 0.7–1.7 length of prehumeral dark spot (PHD) (Figs. 7c, 9e)……*An*. *goeldii*; *An*. *nuneztovari* (*s.s.*) & *An*. *nuneztovari* A
- Wing pale scales, at least on anterior veins, yellowish or grayish; foretarsomere (Ta-I_5_), pale-scaled apically, pale scales varying from yellowish to golden……*An*. *evansae*
- Wing with pale scales white; foretarsomere 5 (Ta-I_5_) variously marked……58
- Foretarsomere 5 (Ta-I_5_) white on apical 0.5……*An*. *dunhami* & *An*. *trinkae*
- Foretarsomere 5 (Ta-I_5_) usually gold to grayish, sometimes dark-scaled on basal 0.5 (Fig. 9c)……*An*. *strodei*
- Hindtarsomeres 3 and 4 (Ta-III_3,4_) with a basal dark-scaled stripe……*An*. *nigritarsis*
- Hind tarsomeres 3 and 4 (Ta-III_3,4_) entirely pale-scaled……60
- Wing vein R_4+5_, predominantly dark-scaled……*An*. *lutzii* & *An*. *guarani*
- Wing vein R_4+5_, predominantly pale-scaled……61
- Wing vein R_4+5_, with 3 dark spots (1 prebasal, 1 median and 1 preapical), clearly separated by 2 pale spots……*An*. *parvus*
- Wing vein R_4+5_, with 2 distinct dark spots (1 prebasal and 1 preapical), region between the 2 dark spots, predominantly pale on dorsal surface of wing……62
- Wing vein CuA_2_ pale-scaled on basal 0.5, dark-scaled on apical 0.5……*An*. *antunesi*
- Wing vein CuA_2_ with 3 distinct pale spots (1 basal, 1 median and 1 apical), interrupted by 2 dark spots interspersed with pale spots (1 prebasal and 1 preapical)……*An*. *pristinus*
- Sternum I (I-S) without longitudinal lines of white scales, rarely with a few sparse scales……64
- Sternum I (I-S) with 2 longitudinal lines of white scales……69
- Palpomere 5 (MPlp_5_) with a dark spot on basal 0.4; scutal scales large; abdominal segments without posterolateral scale-tufts……65
- Palpomere 5 (MPlp_5_) all pale; scutal scales small; abdominal segments with posterolateral scale-tufts at least on segments III-VII……66
- Hindtarsomere 5 (Ta-III_5_) entirely pale-scaled……*An*. *pictipennis*
- Hindtarsomere 5 (Ta-III_5_) with an apical band of dark scales……*An*. *atacamensis*
- Vein C with prehumeral dark spot (PHD) well developed, 3 to 4 times length of humeral pale (HP); anterior mesepimeron with distinct pale scale-patch; upper mesepimeron without pale scales; palpomere 4 (MPlp_4_) with 4 moderately large scale-patches (Figs. 6a, 7b, 12a)……*An*. *darlingi*
- Vein (C) with prehumeral dark spot (PHD) less well-developed, similar in size to humeral pale spot (HP); anterior mesepimeron without a pale scale-patch; upper mesepimeron with a line of pale scales; palpomere 4 (MPlp_4_) without pale scale-spots……67
- Wing with prehumeral dark (PHD) and humeral dark (HD) spots small or absent; hindtarsomere 1 (Ta-III_1_) with a distinct band of apical pale scales; abdominal tergal scales white……*An*. *lanei*
- Wing with prehumeral dark (PHD) and humeral dark (HD) spots more developed, larger; hindtarsomere 1 (Ta-III_1_) without apical pale scales or with a few pale scales not forming a distinct band; abdominal tergal scales differently colored, never white……68
- Abdominal terga II-IV (II-IV-Te) with reddish scales medially, yellowish scales laterally; vein C with prehumeral dark spot (PHD) ≤ 0.5 length of humeral pale spot (HP); hindtarsomere 2 (Ta-III_2_) with a band of dark scales on basal 0.15; interocular space wide, ≥ 0.8 or more diameter of the pedicel……*An*. *sawyeri*
- Abdominal terga II-IV (II-IV-Te) with cream-colored scales medially and some brown scales on mid-apical area; vein C with prehumeral dark spot (PHD) about 0.4–0.8 length of humeral pale spot (HP); hindtarsomere 2 (Ta-III_2_) usually with a band of dark scales on more than basal 0.15; interocular space moderately wide, ≤ 0.8 or less diameter of pedicel……*An. argyritarsis*
- Posterolateral scale-tufts well developed on abdominal tergum II (II-Te): costa (C) with a small sector pale spot (SP); hindtarsomere 2 (Ta-III_2_) with dark basal band 0.3–0.4 length of tarsomeres (Fig. 9a)……*An*. *braziliensis*
- Posterolateral scale-tufts absent from abdominal tergum II (II-Te); costa (C) without a sector pale spot (SP); hindtarsomere 2 (Ta-III_2_) basal dark band variable, ≥ 0.9 length of tarsomere……70
- Hindtarsomere 2 (Ta-III_2_) with basal dark band ≤ 0.5 length of tarsomere, sometimes ≥ 0.63 length of tarsomere……*An*. *marajoara*, *An*. *janconnae* & *An*. *oryzalimnetes*
- Hindtarsomere 2 (Ta-III_2_) with basal band 0.5–0.9 length of tarsomere……71
- Hindtarsomere 1 (Ta-III_1_) with conspicuous apical band of white scales; abdominal terga with posterolateral scale-tufts on segments IV-VII (IV-VII-Te)……*An*. *deaneorum*
- Hindtarsomere 1 (Ta-III_1_) without or with inconspicuous apical band of white scales; abdominal terga with posterolateral scale-tufts on segments III-VII (III-VII-Te) (Figs. 9b, 14)……*An*. *albitarsis*

## Conclusions

Our identification key, based on morphological characters of adult females, can be used to separate South American subgenera and species of the genus *Anopheles*. This key will serve a wide range of users. It will be: (i) reliable to a large degree in that many species can be identified definitively using morphological characters, especially if characters from additional life stages can be included; (ii) cost-effective for many. Morphological identification is still much less expensive and less technology-dependent than molecular methods; (iii) a unique research resource for the identification of specimens to morphospecies, which is needed as a basis for molecular studies. Molecular tools are increasingly effective for enhancing *Anopheles* taxonomy by uncovering similar species, species complexes and sibling species. Identification to morphospecies allows for focus on a subset of individuals rather than having to broadly sample throughout a wide geographical distribution; (iv) a resource for control. Control actions can be justified based on morphological identifications that narrow down to a vector group. Even with the potential of misidentification it is better to assume one is dealing with an effective vector, and that control action is required, rather than to not act at all. This identification key, however, does not allow separation of individual species in a number of informally named groups: i.e. Konderi, Oswaldoi, Nuneztovari, Benarrochi and Albitarsis Complexes, and the Triannulatus and Strodei Groups. In the key these are given species names and designated as “*sensu lato*”. To include component species in future keys, taxonomic studies are needed to name and describe them and to uncover differential characters.

## Data Availability

Specimens used in the current study are deposited and available in the Coleção Entomológica de Referência, Faculdade de Saúde Pública, Universidade de São Paulo (FSP-USP), São Paulo State, Brazil, the US National Mosquito Collection, Smithsonian Institution, Washington, DC, USA (USNMC), and the Facultad de Ciencias Naturales y Exactas de la Universidad del Valle, Colombia.
